# Imatinib and Nilotinib Reverse Multidrug Resistance in Cancer Cells by Inhibiting the Efflux Activity of the MRP7 (ABCC10)

**DOI:** 10.1371/journal.pone.0007520

**Published:** 2009-10-20

**Authors:** Tong Shen, Ye-Hong Kuang, Charles R. Ashby, Yu Lei, Angel Chen, Ying Zhou, Xiang Chen, Amit K. Tiwari, Elizabeth Hopper-Borge, Jiangyong Ouyang, Zhe-Sheng Chen

**Affiliations:** 1 Department of Pharmaceutical Sciences, College of Pharmacy and Allied Health Professions, St. John's University, Queens, New York, United States of America; 2 Department of Dermatology, Xiang Ya Hospital, Central South University, Changsha, China; 3 Fox Chase Cancer Center, Philadelphia, Pennsylvania, United States of America; The Research Institute for Children at Children's Hospital New Orleans, United States of America

## Abstract

**Background:**

One of the major mechanisms that could produce resistance to antineoplastic drugs in cancer cells is the ATP binding cassette (ABC) transporters. The ABC transporters can significantly decrease the intracellular concentration of antineoplastic drugs by increasing their efflux, thereby lowering the cytotoxic activity of antineoplastic drugs. One of these transporters, the multiple resistant protein 7 (MRP7, ABCC10), has recently been shown to produce resistance to antineoplastic drugs by increasing the efflux of paclitaxel. In this study, we examined the effects of BCR-Abl tyrosine kinase inhibitors imatinib, nilotinib and dasatinib on the activity and expression of MRP7 in HEK293 cells transfected with MRP7, designated HEK-MRP7-2.

**Methodology and/or Principal Findings:**

We report for the first time that imatinib and nilotinib reversed MRP7-mediated multidrug resistance. Our MTT assay results indicated that MRP7 expression in HEK-MRP7-2 cells was not significantly altered by incubation with 5 µM of imatinib or nilotinib for up to 72 hours. In addition, imatinib and nilotinib (1-5 µM) produced a significant concentration-dependent reversal of MRP7-mediated multidrug resistance by enhancing the sensitivity of HEK-MRP7-2 cells to paclitaxel and vincristine. Imatinib and nilotinib, at 5 µM, significantly increased the accumulation of [^3^H]-paclitaxel in HEK-MRP7-2 cells. The incubation of the HEK-MRP7-2 cells with imatinib or nilotinib (5 µM) also significantly inhibited the efflux of paclitaxel.

**Conclusions:**

Imatinib and nilotinib reverse MRP7-mediated paclitaxel resistance, most likely due to their inhibition of the efflux of paclitaxel via MRP7. These findings suggest that imatinib or nilotinib, in combination with other antineoplastic drugs, may be useful in the treatment of certain resistant cancers.

## Introduction

Although the clinical use of surgery, radiation and chemotherapy have decreased the recurrence rates of cancer, cellular resistance to chemotherapeutic drugs is a major obstacle in the treatment of cancer [1], [2]. The efficacy of chemotherapy can be limited due to acquired resistance from previous treatment. Consequently, research strategies to circumvent such resistance in cancer cells have become a current focus for the development of novel combinational chemotherapeutic strategies. Both intrinsic and acquired drug resistance can produce multiple changes in various cellular pathways, leading to a decrease in the cytotoxicity, and thus the efficacy, of antineoplastic drugs [3]. Therefore, cancer patients that receive multiple treatments can become increasingly insensitive to chemotherapeutic agents.

One of the primary cellular mechanisms that can produce resistance to antineoplastic therapy involves the efflux of drugs from the cancer cells by specific transmembrane transporters or pumps [4]. These transporter proteins originate from the superfamily of ATP-binding cassette (ABC) transporters that share common structural and functional properties [5]. A number of studies have shown that the majority of the members of the C family of ABC transporters are multidrug resistance proteins (MRPs), which are characterized by cross-resistance to many structurally unrelated drugs [2], [4].

A number of studies suggest that cancer cells that express the ABC C family transporter MRP7/ABCC10 can develop resistance to various chemotherapeutic drugs. For example, human salivary gland adenocarcinoma (SGA) cells that overexpress MRP7 mRNA and the MRP7 protein display significant resistance to vincristine [6]. MRP7 expression has also been immunohistochemically identified in tumor-bearing mice xenografted with human SGA following treatment with vincristine [6]. Furthermore, E_2_17βG, a competitive inhibitor of MRP7 transport, significantly decreased docetaxel accumulation in human SGA cells [6]. Overall, compounds that are inhibitors of MRP7 transport activity attenuate or reverse resistance in cancer cells that express the MRP7 protein.

The MRP7-overexpressing cells confer resistance to several anticancer drugs including paclitaxel, vincristine and vinblastine [7]. Recent papers also reported that MRP7-overexpresssing cells confer resistance to nucleoside analogues and epithilone B [8]. Previously, in our laboratory, we have shown that cepharanthine, a biscoclaurine-derived alkaloid, reversed MRP7-mediated paclitaxel resistance [9].

Tyrosine kinase inhibitors (TKIs) can reverse the resistance of cancer cells to antineoplastic drugs through multiple mechanisms. For example, in human SGA cells, MRP7, P-gp, and MRP1 were all detected after prolonged exposure to vincristine [6]. Recently, we and others have reported that some of the TKIs are potent modulators of ABC transporters, including P-gp and BCRP/ABCG2 [10], [11]. Recent results from our laboratory suggested that nilotinib significantly reverses P-gp- and BCRP-mediated MDR [12].

In this study, one of the main goals was to identify TKI compounds that would reverse MRP7-mediated drug resistance. Consequently, it is possible that TKIs, in combination with other antineoplastic drugs, may be useful in the treatment of cancers that express MDR proteins, including the ABC transporters. An important discovery about TKIs was that certain so-called “small molecule” drugs could inhibit TK activity by competing with ATP for binding to the intracellular catalytic domain of receptor TKs, which produced inhibition of various downstream signaling cascades by autophosphorylation [13]. Interestingly, imatinib, nilotinib and dasatinib are inhibitors of the TK breakpoint cluster region- Abelson (BCR-Abl) and KIT, a class III receptor TK [14]–[18]. The BCR-Abl gene is associated with a dysregulation of TK function and subsequently leads to malignant transformation in chronic myelogenous leukemia (CML) [19], [20]. The recognition of BCR-Abl gene and its corresponding protein has led to the development of small-molecule drugs designed to block the activation of BCR-Abl TKIs through competitive binding at the ATP-binding site [19]. Overall, the primary aim of this study was to determine if the BCR-Abl TKIs could reverse MRP7-mediated MDR.

## Materials and Methods

### 2.1. Cell lines

HEK293 cells and the MRP7 cDNA were generously provided by Dr. Gary Kruh (University of Illinois at Chicago, Chicago, IL). The transfected HEK-MRP7-2 cells and empty vector transfected HEK293-pcDNA3.1 cells were established from HEK293 cells through electroporation [9]. The parental drug-sensitive human epidermoid carcinoma cell line KB-3-1 and its corresponding P-gp-overexpressing cell line KB-C2 were kindly provided by Drs. Gottesman (NCI, Bethesda, MD) and Akiyama (Kagoshima University, Japan), respectively. The KB-C2 cells were established from KB-3-1 cells by exposing them to increasing concentrations of colchicine in a gradual step-wise manner (up to 2 µg/ml) and harvesting the cells that were resistant [20]. All of these cell lines were grown as adherent monolayers in flasks with Dulbecco's modified Eagle's medium (DMEM) supplemented with 10% bovine serum, 2 mM glutamine, 100 units/ml penicillin, and 100 µg/ml streptomycin under standard cell culturing conditions in a humidified incubator containing 5% CO_2_ at 37°C.

### 2.2. Materials

**Figure 1:**
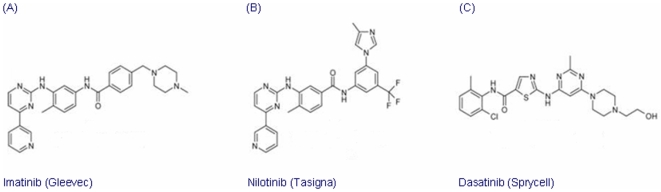
Chemical structures of imatinib (A), nilotinib (B), and dasatinib (C).

DMEM, bovine serum and penicillin/streptomycin were purchased from Hyclone (Logan, UT). Nilotinib (Tasigna®) (Fig. 1B) was obtained as a gift from Novartis pharmaceuticals (Basel, Switzerland). Imatinib (Fig. 1A) and dasatinib (Fig. 1C) were purchased from ChemieTeck Inc. (Indianapolis, IN). Paclitaxel, vincristine, doxorubicin, colchicine, p-aminophenylmethylsulfonyl fluoride, bovine serum albumin, dimethyl sulfoxide (DMSO) and 1-(4,5-dimethylthiazol-2-yl)-3,5-diphenylformazan (MTT), the polyclonal goat antibody against MRP7 (C-19), the monoclonal mouse antibody against P-gp (P7965), the secondary horseradish peroxidase-labeled anti-goat or anti-mouse IgG were purchased from Sigma-Aldrich Chemical Co. (St. Louis, MO). A polyclonal antibody against human MRP1/ABCC1 was kindly provided by Dr. Akiyama (Kagoshima University, Japan) [21]. A monoclonal antibody BXP-34 against BCRP was acquired from Signet Laboratories Inc. (Dedham, MA). [^3^H]-paclitaxel (45 µCi/mmol) was purchased from Moravek Biochemicals (Brea, CA).

### 2.3. Preparation of cell lysates

Confluent monolayer cells in T-25 flask were harvested and rinsed twice with cold PBS. The cell extracts were prepared using the Radioimmunoprecipitation assay buffer [1× PBS, 1% Nonidet P-40, 0.5% sodium deoxycholate, 0.1% SDS, 100 µM p-APMSF, 10 µM leupeptin, and 10 µM aprotinin] for 30 min on ice with occasional rocking followed by centrifugation at 12,000 rpm at 4°C for 15 min. The protein concentrations of cell lysates were determined by the Bradford protein assay [22]. The supernatant containing total cell lysates were collected and stored at −80°C until use.

### 2.4. Immunoblotting

Equal amount of total cell lysates (40 µg) were resolved by 4–12% sodium dodecyl sulfate polycrylamide gel electrophoresis (SDS-PAGE) and electrophoretically transferred onto nitrocellulose membranes [23]. The cell lysates were denatured in a 100°C water beaker for 5 min before loading onto the 4–12% SDS-PAGE. The gel was run in the SDS electrophoresis buffer (25 mM Tris base, 0.192 M glycine, 1% SDS) at 170 V for 2 h. The transfer was performed in a transfer buffer (25 mM Tris base, 0.192 M glycine, PH 8.3) at 30 V for 2 h. The nitrocellulose membrane was then immersed in 5% skim milk to block nonspecific binding for 1 h at room temperature. The membrane was then immunoblotted overnight with primary antibodies (polyclonal MRP7 or monoclonal P-gp and BCRP antibodies at 1∶500 and polyclonal MRP1 at 1∶3,000) at 4°C. The following day, the membrane was washed three times with TBST buffer (0.3% Tris, 0.8% NaCl, 0.02% KCl, 0.05% Tween 20) followed by a three-hour incubation with secondary antibodies of polyclonal anti-MRP7 or monoclonal anti-P-gp at 1∶1000. The protein-antibody complex was measured using an enhanced chemiluminescence detection system (Amersham, NJ). The membrane was then exposed to the film for development. The conventionally used loading control actin was used to detect equal loading in each lane in the samples prepared from cell lysates.

### *2.5. Analysis of drug sensitivity*

Drug sensitivity was analyzed using a slightly modified MTT colorimetric assay [23]. Empty vector transfected HEK293-pcDNA3.1 cells and MRP7-transfected HEK293-MRP7-2 cells were seeded in 96-well plates in triplicate at 5000 cells/well. After incubation in DMEM supplemented with 10% bovine serum at 37°C for 24 h, the antineoplastic drugs were diluted to various concentrations and incubated with the cells continuously for 72 h. The potential inhibitors were added 1 h prior to the addition of the anticancer drugs.

After drug incubation of 72 h, 20 µl MTT (4 mg/ml) was added to each well and the plate was further incubated for 4 h, allowing viable cells to develop from the yellow-colored MTT into dark-blue formazan crystals. Subsequently, the medium was gently removed without agitating the adhesive monolayer of cells, and 100 µl of DMSO was added into each well to dissolve the formazan crystals. The plates were well shaken for 5 min, and an OPSYS microplate reader read the absorbance at 570 nm from DYNEX Technologies Inc. (Chantilly, VA). The degree of resistance was calculated by dividing the IC_50_ for the MDR cells by that of the parental cells, whereas the degree of MDR reversal was calculated by dividing the IC_50_ of the cells with the anticancer drug in the absence of inhibitor by that obtained in the presence of the inhibitor. The concentrations required to inhibit growth by 50% of the control cells were calculated from survival curves using the Bliss method [23].

The antineoplastic drugs used included paclitaxel, vincristine and doxorubicin at varying concentrations up to a final concentration of 10, 1, and 1 µM, respectively. The BCR-Abl TKIs, such as nilotinib and imatinib, were subsequently used at nontoxic concentrations of 1, 2.5 and 5 µM to screen against vincristine and doxorubicin. In this study, we first selected a single nontoxic dose (5 µM) to examine the effects of TKIs on MRP7-mediated resistance to paclitaxel. Once we determined which TKIs had the most significant reversal effect, such as imatinib and nilotinib, we subsequently selected three concentrations (1, 2.5 or 5 µM) for each TKI to determine whether their reversal effects were concentration-dependent to paclitaxel, vincristine and doxorubicin.

### 2.6. Drug accumulation and efflux

The HEK293-pcDNA3.1 parental cells and HEK-MRP7-2 transfected cells were seeded in two T75 flasks and incubated with DMEM supplemented with 10% bovine serum at 37°C. After the cells were 60 to 95% confluent, each inhibitor was added to separate flasks and the cells were incubated for 1 h. The cells were then trypsinized and two aliquots (48×10^6^ cells) from each cell line were suspended in the medium. Subsequently, cells were suspended in the medium containing [^3^H]-paclitaxel at a concentration of 0.1 µM with or without the TKIs nilotinib and imatinib (5 µM) for 1 h at 37°C. One hour later, the incubation medium was replaced by the medium containing the TKIs without paclitaxel. Aliquots (1×10^6^ cells) were collected at various time points (0, 20, 60, and 120 min). The cells were then washed with ice-cold PBS and each sample was placed in scintillation fluid to measure the radioactivity in a Packard TRI-CARB 1900CA liquid scintillation counter from Packard Instrument Inc. (Downers Grove, IL).

### 2.7. Statistical analysis

All experiments were repeated at least three times and the differences were determined by two-tailed Student's t-test. The a priori statistical significance was set at *P*<0.05.

## Results

### 3.1. Expression of MRP7 in HEK293-pcDNA3.1 and HEK-MRP7-2 cells

**Figure 2:**
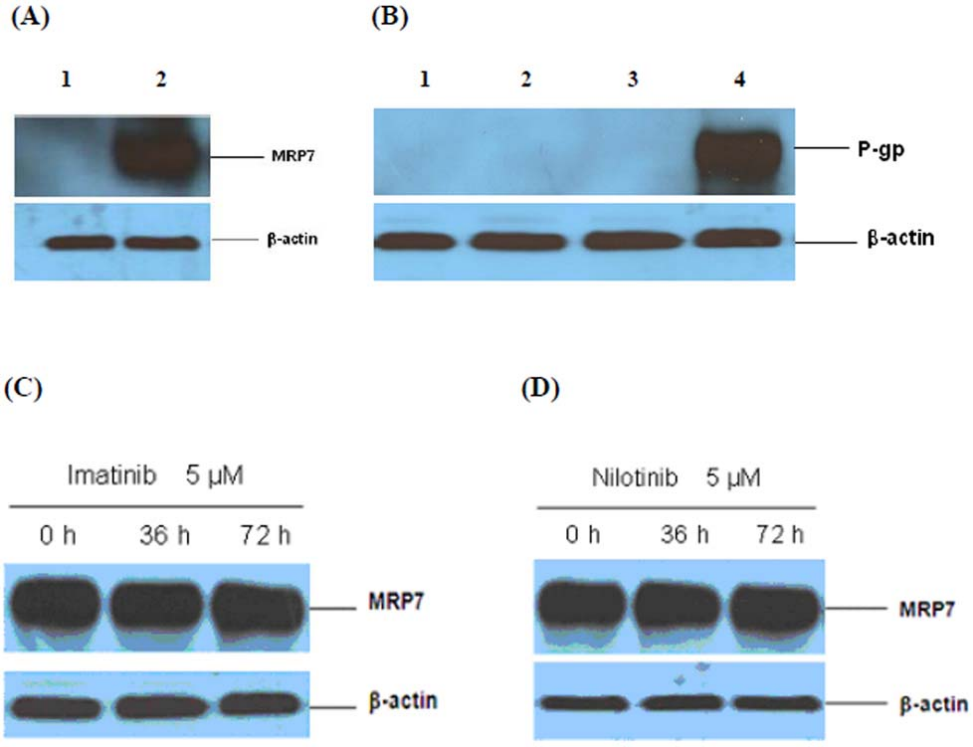
Immunoblot detection of MRP7 and P-gp and the effect of imatinib or nilotinib on MRP7 expression. (A) Expression of MRP7 in HEK293-pcDNA3.1 (lane 1) and MRP7-transfected cells (lane 2). (B) Expression of P-gp in HEK293-pcDNA3.1 (lane 1), HEK-MRP7-2 (lane 2), KB-3-1 (lane 3) and KB-C2 cells (lane 4). (C) Effect of 5 µM of imatinib on the expression level of MRP7 (HEK-MRP7-2) for 36 and 72 h, respectively. (D) Effect of 5 µM of nilotinib on the expression level of MRP7 (HEK-MRP7-2) for 36 and 72 h, respectively. Equal amounts (40 µg protein) of total cell lysate were used for each sample. The nitrocellulose membranes were immunoblotted with primary antibody against MRP7 or actin at 1∶500 dilution, or P-gp at 1∶500 dilution at 4°C overnight, and then incubated with HRP-conjugated secondary antibody at 1∶1000 dilutions at room temperature for 3 h.

In this study, the two cell lines utilized were HEK293 cells transfected with either the MRP7 expression vector or the empty vector control (pcDNA3.1). Immunoblot analysis was performed to detect the expression level of MRP7 protein in the aforementioned lines. MRP7 protein (MW 171 kD) was expressed in HEK-MRP7-2 cells, but not in HEK293-pcDNA3.1 cells (Fig. 2A). P-gp, with a molecular weight of 170 kD, was detected in the positive control KB-C2 cell lines, but not in the negative control KB-3-1, HEK293-pcDNA3.1 or HEK-MRP7-2 cell lines (Fig. 2B). We also conducted Western blot experiments to determine if MRP1 and BCRP were expressed in HEK293-pcDNA3.1 and HEK-MRP7-2 cells. The levels of MRP1 and BCRP in both HEK-MRP7-2 cells and HEK293-pcDNA3.1 cells were undetectable. These findings are important, as any effects observed with the inhibitors are unlikely to be due to their interaction with P-gp, MRP1 and/or BCRP.

To evaluate the effect of imatinib/nilotinib on the expression of MRP7, the HEK-MRP7-2 cells were incubated with 5 µM of imatinib or nilotinib for 36 and 72 h, respectively. The incubation of HEK-MRP7-2 cells with imatinib or nilotinib did not significantly alter the expression of the protein levels of MRP7 at different time points (Fig. 2C and D). This suggests that the effect of the inhibitors on the response of cells to the anticancer drugs is not due to the regulation of MRP7 expression.

### 3.2. Analysis of the drug sensitivity of MRP7-transfected HEK293 cells

To determine the drug resistance profile of MRP7, the sensitivity of HEK-MRP7-2 transfected cells to specific antineoplastic drugs was compared to that of the vector-only control cells, HEK293-pcDNA3.1. The HEK-MRP7-2 cells exhibited a significant higher level of resistance to paclitaxel and vincristine (9.5- and 6.7-fold resistance compare to the control cells, respectively) (Table 1). These results indicated that the HEK-MRP7-2 cell line was able to confer resistance to various antineoplastic drugs, which is consistent with our previous report [9].

**Table 1:** The effect of nilotinib, imatinib and dasatinib on the sensitivity of HEK293-pcDNA3.1 and HEK-MRP7-2 cells to paclitaxel, vincristine and doxorubicin.

	HEK293-pcDNA3.1					HEK-MRP7-2				
Compound	IC_50_	±	SD a (nM)	RF b	DMF c	IC_50_	±	SD a (nM)	RF b	DMF c
**Paclitaxel**	21.85	±	1.89	(1.00)		207.03	±	19.69	(9.47)	
+ Nilotinib 1 µM	10.15	±	0.94	(0.46)	2.15	26.34	±	1.34	(1.21)	7.86 *
+ Nilotinib 2.5 µM	8.61	±	0.32	(0.39)	2.54	15.87	±	0.94	(0.73)	13.05 **
+ Nilotinib 5 µM	7.63	±	0.15	(0.35)	2.86	9.90	±	0.42	(0.45)	20.91 **
+ Imatinib 1 µM	16.17	±	0.77	(0.74)	1.35	44.43	±	2.07	(2.03)	4.66 *
+ Imatinib 2.5 µM	13.70	±	0.82	(0.63)	1.59	22.75	±	2.36	(1.04)	9.10 *
+ Imatinib 5 µM	9.76	±	0.82	(0.45)	2.24	16.63	±	2.36	(0.76)	12.45 **
+ Dasatinib 2.5 µM	19.87	±	0.94	(0.91)	1.10	204.11	±	13.45	(9.34)	1.01
**Vincristine**	9.86	±	1.89	(1.00)		66.38	±	9.69	(6.73)	
+ Nilotinib 1 µM	8.40	±	0.10	(0.85)	1.17	31.73	±	0.91	(3.22)	2.09
+ Nilotinib 2.5 µM	7.60	±	0.32	(0.77)	1.30	9.71	±	1.44	(0.98)	6.84 *
+ Nilotinib 5 µM	6.64	±	2.46	(0.67)	1.48	7.39	±	0.77	(0.75)	8.98 *
+ Imatinib 1 µM	7.33	±	0.75	(0.74)	1.35	27.16	±	2.02	(2.75)	2.44
+ Imatinib 2.5 µM	7.08	±	0.82	(0.72)	1.39	9.62	±	0.36	(0.98)	6.90 *
+ Imatinib 5 µM	7.10	±	0.56	(0.72)	1.39	8.05	±	0.58	(0.82)	8.25 *
+ Dasatinib 2.5 µM	9.19	±	0.88	(0.93)	1.07	62.74	±	7.11	(6.36)	1.06
**Doxorubicin**	30.10	±	0.75	(1.00)		38.45	±	0.36	(1.28)	
+ Nilotinib 1 µM	25.73	±	0.10	(0.85)	1.17	31.73	±	0.91	(1.05)	1.21
+ Nilotinib 2.5 µM	33.77	±	0.32	(1.12)	0.89	36.52	±	1.44	(1.21)	1.05
+ Nilotinib 5 µM	31.36	±	2.46	(1.04)	0.96	33.25	±	1.77	(1.10)	1.16
+ Imatinib 1 µM	31.52	±	0.75	(1.05)	0.95	42.18	±	2.02	(1.40)	0.91
+ Imatinib 2.5 µM	34.14	±	0.82	(1.13)	0.88	42.96	±	0.36	(1.43)	0.90
+ Imatinib 5 µM	32.77	±	0.56	(1.09)	0.92	38.75	±	0.58	(1.29)	0.99
+ Dasatinib 2.5 µM	27.33	±	1.57	(0.91)	1.10	32.47	±	1.33	(1.08)	1.18

a Values represent the mean±SD of at least three independent experiments performed in triplicate.

b Fold resistance was the IC_50_ values for paclitaxel, vincristine, and doxorubicin of HEK293-pcDNA3.1 in the presence of either nilotinib, imatinib or dasatinib, or the transfected cells HEK-MRP7-2 with or without the reversing agents, divided by the IC_50_ values for paclitaxel, vincristine, and doxorubicin of HEK293-pcDNA3.1 cells without the reversing agents. Cell survival was determined by the MTT assay as described in “Section 2”.

c Dose-modifying factor was the ratio of IC_50_ values without reversal agent to the IC_50_ values with reversal agents. * Significantly different from the control transfected as assayed by the Student's t-test (*P*<0.05); ** *P*<0.01. The experiments were repeated at least three times.

### 3.3. The effect of TKIs on sensitivity of MRP7-transfected HEK293 cells to anticancer drugs

**Figure 3:**
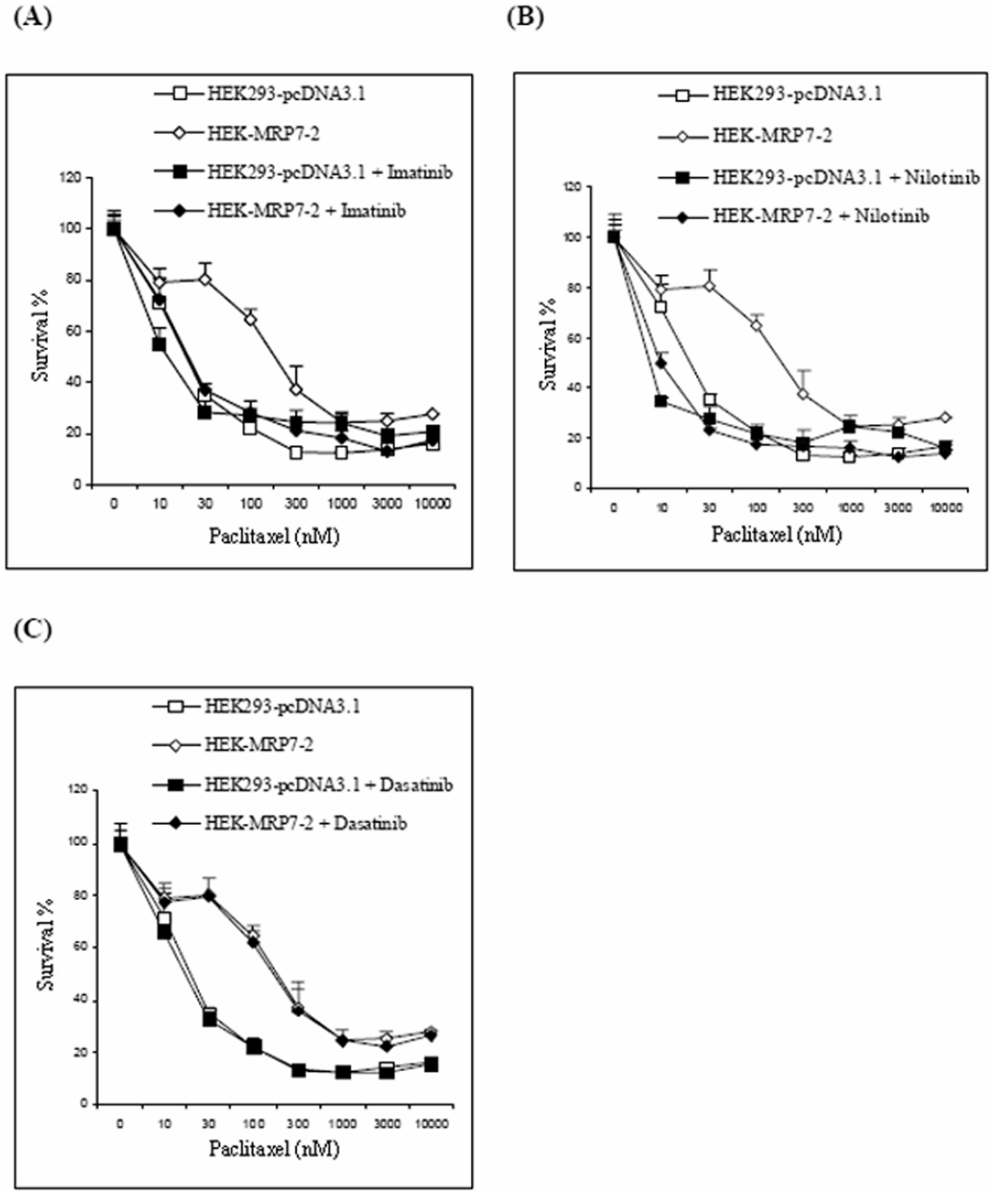
The effect of imatinib (A), nilotinib (B) and dasatinib (C) on the sensitivity of MRP7-transfected HEK293 cells to paclitaxel. Two cell lines, HEK293-pcDNA3.1 and HEK-MRP7-2, are represented as HEK293 and MRP7, respectively. After seeding and culturing cells for 24 h, equal amounts of PBS or the reversal agents were added into HEK293-pcDNA3.1 cells (shown as 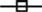 and 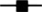, respectively) and HEK-MRP7-2 cells (shown as 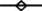 and 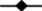, respectively) 1 h before the addition of paclitaxel. The various concentrations of paclitaxel were indicated in the figure. The final concentration for imatinib, nilotinib or dasatinib was 2.5 µM. The above figure shows a representative result for imatinib, nilotinib or dasatinib.

We tested several BCR-Abl TKIs to determine if they could reverse the resistance of HEK293 cells overexpressing MRP7 to the antineoplastic drug paclitaxel and vincristine. The magnitude of reversal produced by the TKIs to paclitaxel was variable (Table 1; Fig. 3). The preincubation of cells with imatinib or nilotinib, at 2.5 µM, significantly reversed the resistance of HEK-MRP7-2 cells to paclitaxel (Table 1, Fig. 3A and 3B). Imatinib and nilotinib produced a 6.9- and 13.0-fold reversal, respectively, of the resistance to paclitaxel. The IC_50_ of paclitaxel in HEK-MRP7-2 cells co-cultured with 2.5 µM of nilotinib was significantly decreased from 207.0±19.7 nM to 15.9±0.9 nM, and this was significantly lower than that of paclitaxel in the control group (21.9±1.9 nM). The resistance to paclitaxel was completely reversed when imatinib was co-incubated with paclitaxel in HEK-MRP7-2 cells. A significantly greater reversal was obtained at the 5 µM (12.4-fold) compared to the 1 µM (4.7-fold) concentration. Imatinib, at 5 µM, also increased the sensitivity of cells to paclitaxel in HEK293-pcDNA3.1 cells by 2.2-fold, although this effect in HEK293-pcDNA3.1 was significantly lower than that in the MRP7 transfected cells (12.5-fold) (Table 1). These results indicated that the BCR-Abl TKIs imatinib and nilotinib significantly attenuated the resistance to paclitaxel mediated by MRP7. Although the co-incubation of HEK293-pcDNA3.1 (control cells) with nilotinib and paclitaxel also enhanced the sensitivity to paclitaxel (a 2.5-fold shift in HEK293-pcDNA3.1 cells), this enhanced sensitivity was significantly lower than that determined for the MRP7 transfected cells (Table 1; Fig. 3C). In contrast, another BCR-Abl TKI, dasatinib at 2.5 µM, did not significantly enhance paclitaxel sensitivity in either HEK293-pcDNA3.1 or HEK-MRP7-2 cells (Table 1, Fig. 3C).

In addition to paclitaxel, we also examined the effect of the selected TKIs to sensitize cells to another anticancer drug, vincristine. Similar to the findings with paclitaxel, nilotinib and imatinib (1, 2.5 and 5 µM) significantly reversed MRP7-mediated vincristine resistance (2.1-, 6.8- and 9.0-fold, respectively, for nilotinib; 2.4-, 6.9- and 8.2-fold, respectively, for imatinib) in a concentration-dependent manner (Table 1). We also examined the response of MRP7-transfected cells to another anticancer drug, doxorubicin, in the presence of imatinib, as doxorubicin is not a substrate of MRP7 [23]. Our results indicated that imatinib (1, 2.5 and 5 µM) did not significantly sensitize the response of control parental HEK293-pcDNA3.1 cells, or reverse resistance in HEK-MRP7-2 transfected cells, (Table 1) to doxorubicin. This indicates that the response to these TKIs was specific for MRP7, as doxorubicin is not a substrate for MRP7 and thus would not mediate doxorubicin efflux. Doxorubicin is a substrate of P-gp, MRP1 and BCRP, but neither imatinib nor nilotinib significantly increased doxorubicin sensitivity of HEK-MRP7-2 cells, suggesting that P-gp, MRP and BCRP do not significantly contribute to the drug resistance of HEK-MRP7-2.

Overall, imatinib and nilotinib significantly reversed MRP7-mediated resistance to paclitaxel and vincristine, but not doxorubicin. Furthermore, this reversal was concentration-dependent (Table 1; Fig. 3). Although an increase in the sensitization of the HEK293-pcDNA3.1 control cells occurred upon exposure to nilotinib or imatinib, this effect was significantly lower than that determined for the HEK-MRP7-2 transfected cells (Table 1; Fig. 3).

### 3.4. The effects of imatinib and nilotinib on the intracellular accumulation and efflux of [^3^H]-paclitaxel

**Figure 4:**
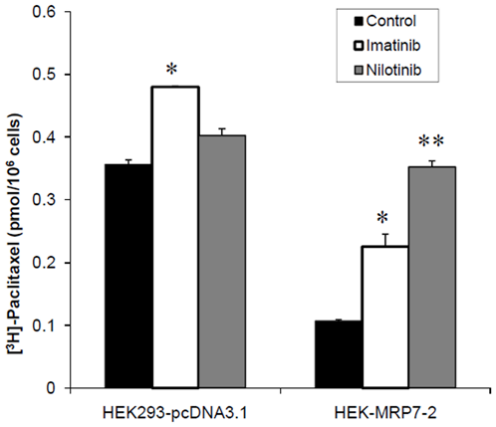
The effect of imatinib (A) or nilotinib (B) on the accumulation of [^3^H]-paclitaxel in HEK293-pcDNA3.1 and HEK-MRP7-2 cells. The intracellular paclitaxel accumulations in HEK293-pcDNA3.1 and HEK-MRP7-2 cells were measured after the incubation with 0.1 µM paclitaxel. Intracellular accumulation of paclitaxel in HEK293-pcDNA3.1 cells in the absence of imatinib and nilotinib were shown in the left bars (▪). Intracellular accumulation of paclitaxel in the presence of 5 µM of imatinib in HEK293-pcDNA3.1 cells was shown on the right (□). Intracellular accumulation of paclitaxel in HEK-MRP7-2 cells in the presence of 5 µM of nilotinib was shown on the right (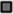). Each column represents the means (±SD). All experiments were done in triplicate. * p<0.05, Student's t test.

In order to determine the mechanism by which imatinib and nilotinib surmount or reverse MRP7-mediated paclitaxel resistance, their effect on the accumulation of [^3^H]-paclitaxel in MRP7-transfected cells was examined. The intracellular concentration of [^3^H]-paclitaxel in HEK-MRP7-2 cells was 30% of that accumulated by the HEK293-pcDNA3.1 cells (Fig. 4). The accumulation of paclitaxel was significantly enhanced (1.9-fold) in HEK-MRP7-2 cells (P<0.05) following incubation of cells with either imatinib or nilotinib at a concentration of 5 µM. In HEK293-pcDNA3.1 cells, imatinib, but not nilotinib, had resulted in a slight increase in the intracellular concentration of [^3^H]-paclitaxel, but this sensitization was modest compared to the effect of imatinib in HEK-MRP7-2 cells.

**Figure 5:**
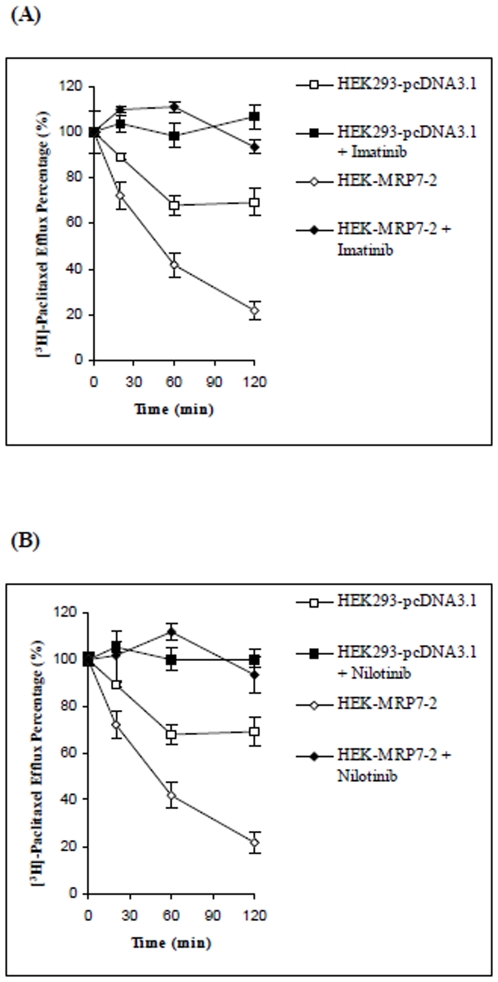
The effect of imatinib (A) or nilotinib (B) on the paclitaxel efflux in HEK293-pcDNA3.1 and HEK-MRP7-2 cells. The percentage of the paclitaxel released was plotted as a function of time. After 1 h of incubation of the TKIs, [^3^H]-paclitaxel was co-incubated in HEK293-pcDNA3.1 with TKI (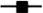) or without TKI (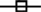), and meanwhile in HEK-MRP7-2 cells with TKI (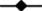) or without TKI (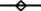). Cells were washed and re-incubated in the paclitaxel-free medium. At the time points of 0 min, 20 min, 60 min and 120 min, the cells were collected and the levels of [^3^H]-paclitaxel were determined by scintillation counting. The values at 0 min of drug efflux were set as 1 for comparison to values measured from other time points. Each point represents the means (±SD) of three separated experiments done using triplicate samples.

Based on the above results, it is possible that the increase in intracellular paclitaxel produced by imatinib and nilotinib could be due to: (1) a decrease in the efflux of paclitaxel and/or, (2) an increase in the uptake of paclitaxel. Therefore, the next experiment was conducted to determine if the increase in paclitaxel accumulation produced by imatinib and nilotinib was due to an inhibition of paclitaxel efflux. HEK-MRP7-2 cells and HEK293-pcDNA3.1 cells were incubated with paclitaxel and a time course for intracellular drug accumulation was determined (Fig. 5). As expected, HEK-MRP7-2 cells released a significantly higher percentage of accumulated paclitaxel compared to HEK293-pcDNA3.1 cells, and the amount of paclitaxel that was effluxed increased with time. The paclitaxel accumulation at 0 min of drug efflux was set as 1. At 60 min, in cells incubated in drug-free medium, ∼60% of the accumulated paclitaxel was exported from HEK-MRP7-2 cells in the absence of imatinib or nilotinib. In contrast, almost all of the paclitaxel was present inside the HEK-MRP7-2 cells incubated with imatinib or nilotinib at a final concentration of 5 µM over different time periods. For the control cells, the concentration of paclitaxel subjected to efflux also increased with time, but the level of efflux was only slightly less than that observed in cells transfected with MRP7. In contrast, in the HE293-pcDNA3.1 cells, ∼30% of the paclitaxel was released after 60 min in the absence of the test compounds. The incubation of the HEK293-pcDNA3.1 cells with imatinib or nilotinib (5 µM) also inhibited the efflux of paclitaxel, although the magnitude of the effect was significantly lower than that observed for the HEK-MRP7-2 cells.

## Discussion

This study was the first to identify that the BCR-Abl TKIs imatinib and nilotinib, but not dasatinib, could reverse MRP7-mediated MDR in a concentration-dependent manner.

HEK293 cells transfected with the MRP7 recombinant gene HEK-MRP7-2, and HEK293-pcDNA3.1 cells transfected with empty vector, were used for the tests of MRP7 efflux function. These transfected cell lines have previously been used in studies designed to determine the effects of cepharanthine on the reversal of MRP7-mediated resistance to paclitaxel [9]. In this study, Western blot analysis confirmed that the transfection of cells with MRP7 was successful, as MRP7 proteins were expressed only in HEK-MRP7-2 cells, and not in HEK293-pcDNA3.1 cells (Fig. 2). The cell lines were exposed to the exact same experimental conditions and procedures, and were cultured with the same antineoplastic drugs for the same incubation time. It may be argued that the MRP7-transfected cells expressed other proteins, in addition to MRP7, such as P-gp, that may have contributed to MDR. However, Western blot analysis indicated that neither the HEK293-pcDNA3.1 control cell line, nor the HEK-MRP7-2 transfected cell line, expressed detectable levels of P-gp (Fig. 2), which can also mediate MDR. In addition, MRP1 and BCRP were undetectable in both control HEK293-pcDNA3.1 and transfected HEK-MRP7-2 cells through Western blot analysis (data not shown). Therefore, these results indicate that the HEK-MRP7-2 transfected cell line specifically expressed MRP7, but not P-gp, MRP, or BCRP.

Previously, it has been reported that cepharanthine and nilotinib significantly reversed P-gp-mediated MDR in HEK-MRP7-2 cells [9] and BCRP-overexpressing cell lines [24], respectively. Since MRP7 and P-gp are similar in structure and functional activity, we designed experiments to determine if nilotinib could reverse MRP7-mediated drug resistance to paclitaxel. Owing to the structural similarity between MRP7 and P-gp, an earlier study indicated that some P-gp inhibitors were able to significantly reverse paclitaxel resistance in HEK-MRP7-2 cells overexpressing MRP7 [9]. We found that nilotinib (2.5 µM) significantly sensitized MRP7-transfected HEK293 cells to paclitaxel as it markedly decreased the IC_50_ of paclitaxel in MRP7-trasnfected cells, compared to control cells. Although nilotinib is an inhibitor of P-gp [24], this is not a confounding factor in our experiments as the P-gp protein is not expressed in either HEK293-pcDNA3.1 or HEK-MRP7-2 cells (Fig. 2).

In this study, we found that specific TKIs: 1) significantly decreased resistance to paclitaxel in MRP7-overexpressing cells (Table 1; Fig. 3), 2) did significantly sensitize the response to paclitaxel in the empty vector transfected cells, but the effect was significantly lower than that in the MRP7 transfected cells (Fig. 3), and 3) did not significantly inhibit or induce MRP7 expression (Fig. 2C and D). Overall, these findings tentatively suggest that of the TKIs used in this study, imatinib and nilotinib are capable of reversing MRP7-mediated resistance by inhibiting the function of MRP7. Among the TKIs that were tested against paclitaxel, the BCR-Abl TKIs imatinib and nilotinib at 5 µM completely reversed MRP7-mediated paclitaxel resistance by a magnitude of 12.5- and 21.0-fold, respectively (Table 1). In contrast, another BCR-Abl TKI, dasatinib, produced no significant reversal of MRP7-mediated paclitaxel resistance (Table 1; Fig. 3C). Currently, the explanation for the difference between dasatinib compared to nilotinib or imatinib remains to be determined. One potential approach that may yield insight into this issue would be that of structural-activity relationship (SAR). Based on their chemical structures (Fig. 1), dasatinib lacks a 2-phenylaminopyrimidine ring that is present in the structures of nilotinib and imatinib. It is possible that the absence of this ring in dasatinib could be an important factor that differentiates dasatinib activity from that of either nilotinib or imatinib. Detailed structure-function studies will be required to delineate the SAR for the interaction of these TKIs with MRP7-overexpressing cells. In addition, it was suggested that the HEK293-pcDNA3.1 cells were also sensitized by co-administering nilotinib or imatinib (Table 1; Fig. 5). Such sensitization could be due to possible endogenous efflux pumps in the HEK293-pcDNA3.1 cells.

In order to extend the findings obtained with paclitaxel, we examined the effect of specific TKIs on the response of MRP7-expressing cells to another antineoplastic drug, vincristine, which is a substrate for MRP7 [23]. Our results indicated that both nilotinib and imatinib (2.5 µM) completely reversed MRP7-mediated vincristine resistance by a factor of 6.8- and 6.9-fold, respectively (Table 1). Thus, nilotinib and imatinib can significantly attenuate MRP7-mediated resistance to not only paclitaxel, but also to vincristine. As an additional control, we examined the effect of nilotinib and imatinib on the response of MRP7-transfected cells to doxorubicin, which is not a substrate for MRP7 [23]. The results indicated that neither nilotinib nor imatinib (1, 2.5, and 5 µM) significantly altered the response of cells to doxorubicin (Table 1). This finding suggests that the actions of the TKIs in reversing MRP7-mediated resistance are due to a specific effect on the MRP7 pump.

It is well established that MRP7, P-gp and MRP1 are all drug efflux pumps responsible for the extracellular transport of a variety of antineoplastic drugs. Consequently, when these pumps are present in the tumor cells concurrently, each of the pumps contributes to the efflux and decrease of intracellular drug concentrations. This latter action ultimately leads to drug levels that are no longer cytotoxic, leading to failure of therapy. For instance, nilotinib has been identified as an inhibitor of P-gp and the BCRP efflux pumps [24]. In the current study, it has also been identified as a reversal agent for MRP7-mediated resistance to paclitaxel. Our findings, provided that they can be translated to *in vivo* function, suggest that specific TKIs may be useful in treating cancer cells that have become resistant to treatment as a result of MRP7 overexpression. The systemic exposures achieved at the standard recommended doses of both imatinib (steady state C_max_/C_min_ 5.2 and 2.5 µM at 400 mg q.d.) and nilotinib (steady state C_max_/C_min_ 4.0 and 1.8 µM at 400 mg b.i.d.) are similar to the concentrations that reversed drug resistance in the present study [25], [26]. Thus, it is possible that TKIs such as those used in this study could be useful chemosensitizing drugs in the clinic for specific patients. Clearly, *in vivo* studies are needed to evaluate the effects of specific TKIs on the resistance of cancer cells to antineoplastic drugs.

Future experiments, based on the current findings, include the: 1) determination of the effects of newly synthesized TKIs to reverse MRP7-mediated drug resistance, and 2) analysis of the effects of TKIs on the efflux activity of other ABC transporters.

In summary, the results of this study are the first to indicate that the BCR-Abl TKIs imatinib and nilotinib, but not dasatinib, at concentrations that did not produce cytotoxicity, significantly reversed MRP7-mediated MDR to paclitaxel and vincristine. Furthermore, these TKIs might be inhibitors for multiple MDR efflux pumps, such as P-gp and BCRP for nilotinib [22]. In addition, it is likely that imatinib and nilotinib reverse MDR in the HEK293-MRP7 transfected cells by inhibiting the efflux activity of the MRP7 (ABCC10) transporter. Provided these results can be extended to human cancer cells, they suggest that the TKIs could be useful in treating patients that exhibit resistance to paclitaxel or vincristine as a result of MRP7. Therefore, TKIs may be useful modifiers of MDR in cancer cells that overexpress P-gp, BCRP and/or MRP7 [22].
