# Correction: Imatinib and Nilotinib Reverse Multidrug Resistance in Cancer Cells by Inhibiting the Efflux Activity of the MRP7 (ABCC10)

**DOI:** 10.1371/annotation/e57b4610-9029-48db-9d57-5cc0fa35b8ac

**Published:** 2009-11-12

**Authors:** Tong Shen, Ye-Hong Kuang, Charles R. Ashby, Yu Lei, Angel Chen, Ying Zhou, Xiang Chen, Amit K. Tiwari, Elizabeth Hopper-Borge, Jiangyong Ouyang, Zhe-Sheng Chen

A sentence was omitted from Section 2.2 of the Methods . The corrected paragraph should read: DMEM, bovine serum and penicillin/streptomycin were purchased from Hyclone (Logan, UT). Nilotinib (TasignaR) (Fig. 1B) was obtained as a gift from Novartis pharmaceuticals (Basel, Switzerland). Imatinib (Fig. 1A) and dasatinib (Fig. 1C) were purchased from ChemieTeck Inc. (Indianapolis, IN). Paclitaxel, vincristine, doxorubicin, colchicine, p-aminophenylmethylsulfonyl fluoride, bovine serum albumin, dimethyl sulfoxide (DMSO) and 1-(4,5-dimethylthiazol-2-yl)-3,5-diphenylformazan (MTT), the monoclonal mouse antibody against P-gp (P7965), the secondary horseradish peroxidase-labeled anti-goat or anti-mouse IgG were purchased from Sigma-Aldrich Chemical Co. (St. Louis, MO). A polyclonal goat antibody against human MRP7 (D-19) was purchased from Santa Cruz Biotech Inc.<br/>(Santa Cruz, CA). A polyclonal antibody against human MRP1/ABCC1 was kindly provided by Dr. Akiyama (Kagoshima University, Japan) [21]. A monoclonal antibody BXP-34 against BCRP was acquired from Signet Laboratories Inc. (Dedham, MA). [3H]-paclitaxel (45 &mu;Ci/mmol) was purchased from Moravek Biochemicals (Brea, CA).

